# Microbiome Against the Background of the Complex Aetiology in Sarcoidosis—What Do We Already Know?

**DOI:** 10.3390/life15071069

**Published:** 2025-07-04

**Authors:** Maciej Szymański, Katarzyna Góralska, Ewa Brzeziańska-Lasota

**Affiliations:** 1 „Szkoła Orłów”, Project by Academic Career Office, Medical University of Lodz, 90-752 Lodz, Poland; maciej.szymanski2@student.umed.lodz.pl; 2 Department of Biology and Parasitology, Chair of Biology and Medical Microbiology, Medical University of Lodz, 90-752 Lodz, Poland; 3 Department of Biomedicine and Genetics, Chair of Biology and Medical Microbiology, Medical University of Lodz, 90-752 Lodz, Poland; e.brzezianska@umed.lodz.pl

**Keywords:** lung–gut axis, microbiome, sarcoidosis

## Abstract

Sarcoidosis is a multi-organ, systemic disease of immunological origin. While its aetiology is unknown, it is believed to be influenced by genetic susceptibility, environmental factors, and autoimmunity. Recent research on the development and progression of sarcoidosis has focused increasingly on the role of microorganisms. Changes in the respiratory tract microbiome, and hence the physiology of the respiratory tract, are believed to influence the course of sarcoidosis; this is not unlikely, as research indicates that the state of the microbiota inhabiting individual ontocenoses, such as the intestines or blood, may be related to the health of the entire body. This review therefore discusses the microbiological factors that are believed to be involved in the development of the disease; however, as the aetiological factors of sarcoidosis are diverse and are based on highly complex mechanisms, our analysis is restricted to only the most likely factors.

## 1. Introduction

Sarcoidosis is a multisystem, systemic disease. While it is known to have an immunological background, its aetiology remains otherwise unclear. It is characterised by the formation of non-necrosing granulomas consisting mostly of epithelioid and multinucleated giant cells surrounded by a layer of T CD4+ cells, together with lesser populations of T CD8+ and B cells [1]. While in over 90% of sarcoidosis cases, these lesions are present in the lungs and thoracic lymph nodes, though they may also be found in the liver, spleen, eyeballs, skeletal muscles, heart muscle, skin, nervous system, and salivary glands [1,2]. While the course of the disease varies significantly between patients, sarcoidosis is typically asymptomatic or demonstrates non-specific symptoms. It can present itself as Löfgren’s syndrome—an acute form that consists of erythema nodosum, hilar lymphadenopathy, fever, and arthritis and tends to resolve spontaneously—or as a chronic form that results in fibrotic changes and dysfunction of the involved organs [3]. Diagnosis is based on a combination of clinical presentation and histopathologic findings with diagnostic imaging, as well as the confirmation of granulomas in suspected organs and exclusion of other similar diseases, e.g., tuberculosis or lymphoma [2].

Lung sarcoidosis is classified according to the five-grade Scadding criteria based on radiology findings [2]. As sarcoidosis tends to self-resolve in over two-thirds of cases, patients with mild symptoms (mostly stage I of lung sarcoidosis) usually require only medical supervision. However, patients with poor prognosis, i.e., with progressing lung sarcoidosis or with granulomas present in the heart, eyes, or nervous system may receive pharmacologic treatment with corticosteroids or immunosuppressants (methotrexate, azathioprine, or anti-TNF-α biological drugs, such as infliximab) [2,4,5].

The causes of sarcoidosis are not well understood, although genetic susceptibility, environmental factors, and autoimmunity may have a part in the development of this disease. However, recent research has focused on the involvement of microorganisms in the development and progression of sarcoidosis. Particular attention has been paid to the contribution of the microbiome, i.e., the set of microorganisms and their genetic material occurring in a specific habitat at a specific time [6]. Molecular techniques indicate that the state of the airway microbiome may significantly influence airway physiology, and that changes in the airway microbiome may be indicative of disorders, including sarcoidosis [5,7,8].

The discovery that a particular microbiota may influence the microorganisms inhabiting other ontocenoses suggests that natural intestinal commensals may shape the health of the entire organism. The possibility of blood-borne transfer of microorganisms originating from the intestine may be of significance here, indicating the important role of the so-called blood microbiome [9,10]. This review discusses the factors involved in the development of sarcoidosis; however, as these causes are diverse and its mechanisms are most likely highly complex and dependent on several factors, its scope is restricted to those that are widely believed to play the greatest roles. These can include genetic factors, environmental abiotic risk factors, and environmental infectious microbial agents, i.e., infections contributing to sarcoidosis and the microbiome.

## 2. Epidemiology

According to the 2021 Global Burden Disease, the combined burden of interstitial lung disease and lung sarcoidosis is estimated to be 4 042 150 DALY [11]. Epidemiological data indicate that most patients are aged between 20 and 50, with a slight predominance of women; a second peak has also been noted in those over their 50s. Annual incidence is about 10 cases per 100,000, but it varies from 1 to 15 per 100,000 depending on the population analysed, with the highest incidence rate noted in Northern Europe (15 cases per 100,000) and North America (10 cases per 100,000). Least susceptible are Eastern Asian populations, with an incidence of around 0.5–1 cases per 100,000 [12,13]. Additionally, the incidence differs between geographic regions, being highest in the populations of Scandinavia and northern Japan, and lowest among those living in the Caribbean, Mediterranean, and Taiwan [14,15,16]. Some researchers also report seasonal changes in the incidence of sarcoidosis in certain geographic regions, which may result from the present environmental factors [17].

## 3. Etiopathogenesis of Sarcoidosis

### 3.1. Genetic Factors

Individual genetic predispositions are believed to influence the development of sarcoidosis by modulating the immunological response to environmental factors [18]. In particular, genes from the VI chromosome Human Leukocyte Antigen (HLA) region, coding for the Major Histocompatibility Complex (MHC), have been directly associated with the etiopathology of sarcoidosis. A meta-analysis by Sivalokanathan [19] identified 27 genes from this region, among which 4 variants were protective (HLA-DQA1*0301, HLA-DQB1*0302, HLA-DRB1*0101, HLA-DRB1*04), 7 were associated with the chronic course of sarcoidosis (HLA-DQA1*0505, HLA-DQB1*0601, HLA-DQB1*0602, HLA-DQB1*1501, HLA-DRB1*14, HLA-DRB1*15, HLA-DRB1*1101), and 4 were associated with Löfgren syndrome (HLA-B*51, HLA-DQA1*0501, HLA-DQB1*0201, HLA-DRB1*03) [19]. Sarcoidosis is characterised by an over-exaggerated Th-1 response, which has been attributed to mutations in *HLA* genes that may influence the intensity of the T cell reaction to presented antigens. Studies also highlight the importance of various genes in the HLA region, such as *BTNL-2* that negatively regulates T cell activation, *HLA-DRB1*, which is a part of MHC class II, and *HLA-B*, which is a part of MHC class I [18,20]. The disease course is also influenced by genes outside of the HLA region, such as *ANXA11* and *XAF1*, whose variants may increase the durability of granulomas by disturbing cell apoptosis [19].

The influence of genetics is further reflected in the observed variation in incidence between different populations, as well as the heredity of sarcoidosis [14]. Patients with sarcoidosis demonstrate significant changes in their genome and gene expression compared to individuals without sarcoidosis, particularly the genes responsible for protein modification, kinase cascade action, and phosphorylation processes. Compared to healthy individuals, sarcoidosis patients demonstrate changes in the regulation of intra- and extracellular signalling pathways associated with *inter alia* antigen presentation, activation and differentiation of immune cells, cytokine response, and cell signalling associated with T and B cell receptors [6].

### 3.2. Environmental Abiotic Risk Factors

One supposed trigger of sarcoidosis is believed to be exposure to non-infectious environmental factors. For example, inhalation of silica dust is associated with above-average incidence. Also, patients with silicone implants are more prone to developing sarcoidosis, with silica being found inside the granulomas and the lesions receding after implant removal [21]. Another triggering factor may be components of smoke and dust, which promote the activation of macrophages in the lungs. Developing and progressing sarcoidosis has been associated with exposure to smoke and dust generated during combustion, as confirmed by numerous cases of sarcoidosis among firefighters participating in rescue operations after the 2001 attack on the World Trade Centre [22]. Researchers also highlight the similarities between sarcoidosis and chronic beryllium disease, or berylliosis, as both diseases involve the Th cell response to the antigens presented by MHC proteins [23].

Sarcoidosis is also significantly common in obese and overweight patients. This is probably connected to the stronger reactions to antigens occurring in those patients, which may facilitate chronic inflammation and granuloma formation. In such cases, the immune system is believed to demonstrate increased activity due to elevated levels of pro-inflammatory leptin produced by adipocytes; this mechanism has also been implicated in the pathomechanisms of various other diseases [24,25]. However, it has not been confirmed that increased BMI is associated with a higher risk of sarcoidosis; indeed, a recent study suggests that obesity-related obstructive sleep apnea may be associated with a lower risk of sarcoidosis [26].

Interestingly, despite being a common risk factor in many other diseases, smoking may serve as a protective factor against sarcoidosis, with a low incidence rate of sarcoidosis noted among tobacco smokers. The fact that smoking has a suppressive effect on Th cells and macrophages may suggest that sarcoidosis is caused in part by excessive activity of the immune system [27]. However, the literature regarding the effect of smoking on the macrophage population is somewhat contradictory, as there is clear evidence that all smokers have significantly higher numbers of macrophages in their lungs. A meta-analysis conducted by Dehara et al. [28] indicates that while non-smokers are more prevalent among patients with sarcoidosis, there is still no clear evidence that smoking inhibits the development of the disease. The authors point out that a significant part of research to date is burdened with high heterogeneity and low quality, which may significantly affect its interpretation [28].

### 3.3. Environmental Infectious Microbial Agents

#### 3.3.1. Infection as Potential Factors Contributing to the Development of Sarcoidosis—Microorganisms as a Triggering Factor

The immunopathogenesis of sarcoidosis is often associated with the *immune paradox*, understood as an apparent inconsistency between peripheral anergy and excessive inflammatory response in affected organs [1,2]. Several distinct immunological mechanisms are thought to contribute to its development. These include an increased proportion of regulatory T cells (Tregs) in peripheral blood, elevated levels of activated CD4+ and CD8+ T cells in bronchoalveolar lavage fluid (BALF) and at sites of granulomatous inflammation, upregulated expression of T helper 1 (Th1) chemokines and chemokine receptors on CD4+ T cells in BALF, and an increased CD4+:CD8+ T cell ratio in regions of localised granulomatous inflammation [29].

**Figure 1:**
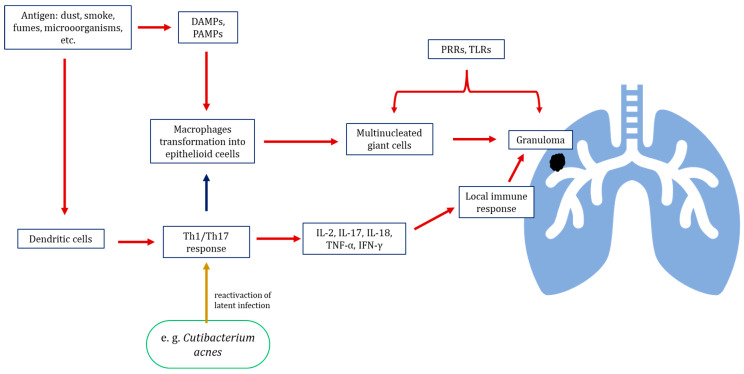
Pathway of immune response activation leading to granuloma development in sarcoidosis. Stimulation by triggering factor stimulates the transformation of macrophages into epithelioid cells, which combine into multinucleated giant cells forming the granuloma core. Th1 and Th17 lymphocytes activated by dendritic cells surround macrophages forming a granuloma core; they also stimulate a local immunological reaction through production of pro-inflammatory cytokines. Lymphocyte activation may additionally be influenced by the reactivation of latent intracellular microorganisms, such as *Cutibacterium acnes*. DAMPs—Danger-Associated Molecular Patterns; PAMPs—Pathogen-Associated Molecular Patterns; PRRs—Pattern Recognition Receptors; TLRs—Toll-like receptors.

A range of external factors, such as components of smoke, fumes, and dust, microorganisms or their metabolites can cause abnormal immunological reactions in the development of sarcoidosis; their mechanisms involve non-specific responses to both Danger- Associated Molecular Patterns and Pathogen-Associated Molecular Patterns (DAMPs and PAMPs), such as human or bacterial heat shock proteins and bacterial metabolites, as well as an adaptive response to various antigens, most of which are exogenous [29,30]. Under the influence of PAMPs and DAMPs, activated macrophages are transformed into epithelioid cells and fuse into multinucleated giant cells that become the foundation of the granuloma core; this is believed to be driven by Pattern Recognition Receptors (PRRs) with Toll-like receptors (TLRs) and stimulated by pro-inflammatory cytokines released by other cells [31]. Lymphocytes are activated by antigen-presenting cells, such as dendritic cells, which promote their differentiation into T helper 1 (Th1) and T helper 17 (Th17) subsets. These effector cells sustain and amplify the local immune response by secreting pro-inflammatory cytokines, including interleukin-2 (IL-2), interleukin-17 (IL-17), tumour necrosis factor-alpha (TNF-α), and interferon-gamma (IFN-γ) (Figure 1). During the development of the disease, lymphocytes form an outer layer for the macrophages constituting the granulomas [32,33]. Meanwhile, the functionality and quantity of regulatory T cells is decreased to the point they are unable to terminate the immune response [34]. This prolonged response damages the surrounding tissue and causes fibrosis, which plays a key role in the complications and deaths associated with sarcoidosis [35].

Studies confirm that an important role in the development of sarcoidosis is played by microorganisms, with microorganism cells, their genetic material, and metabolites being observed in bioptates. Sarcoidosis is most commonly associated with bacteria of the *Mycobacterium* genus (especially *Mycobacterium tuberculosis*) and *Cutibacterium acnes* (formerly *Propionibacterium acnes*) [1,16,36]. These microorganisms provide foreign substances that induce and sustain the immune response or influence the functionality of the immune system; for example, mycobacterial heat shock proteins (mTB-hsps), superoxide dismutase A, and mycobacterial catalase-peroxidase are believed to be responsible for granuloma formation [37]. Akata et al. [38] reported a compelling case involving a 72-year-old patient diagnosed with sarcoidosis. By using Sanger 16S ribosomal RNA sequencing, the authors discovered the presence of *Cutibacterium acnes* and *Streptococcus gordonii* clones within the granulomatous lesions. They implied that these bacterial species may have induced inflammatory cytokine production. *C. acnes* may impact the activity of immune cells by producing short-chain fatty acids (SCFAs) with immunomodulatory properties, such as formic acid, acetic acid, butyric acid, and valeric acid [16]. Some evidence also suggests that *Borrelia* and *Rickettsia* spp. as well as some viruses, e.g., Epstein–Barr virus, Human Herpesvirus, or Retrovirus, may also influence sarcoidosis; however, the body of evidence is small [39].

*C. acnes* are Gram-positive rods that belong to the natural human microbiota, especially on the skin and have been associated with acne vulgaris. Increasing evidence indicates the participation of *C. acnes* in the development of granulomas during sarcoidosis [36,40]. The species has been isolated from 92% of patients with active sarcoidosis; in addition, immunohistochemical analyses revealed the presence of *C. acnes* in 88% of granulomas in the lymph nodes and 74% of granulomas in the lungs [36]. The species has been found to promote Th1 and Th17 immune responses by inducing the production of cytokines such as IL-17, IFN-γ, IL-6, TNF-α, IL-12, and IL-18 [40]. Th1 and Th17 lymphocytes, alongside dendritic cells and macrophages, are the main cells that build granulomas [29]. *C. acnes* cells can circulate extracellularly or, in the case of latent infection, remain intracellularly within macrophages. This latent invasion can be reactivated in individuals with Th1-related hypersensitivity, which promotes the development of granulomas in tissues [36]. It has also been found that in patients with sarcoidosis, *C. acnes* induces increased local production of immunoglobulins IgG and IgA [40].

Numerous case studies indicate considerable similarities between sarcoidosis and tuberculosis with regard to their clinical characteristics; the two diseases have also been found to co-occur in some cases, which may contribute to misdiagnosis [41,42,43]. Furthermore, bacteria of the genus *Mycobacterium*, which are responsible for tuberculosis, leprosy, and Buruli ulcer, have also been associated with sarcoidosis. However, tuberculosis is characterised by a different picture of immune activation to sarcoidosis: tuberculosis exhibits M1 and M2-like macrophage activation with a high level of TGF-β production and low IFN-γ, while sarcoidosis demonstrates mainly M1-like macrophages, no TGF-β production, and an increased level of IFN-γ [1]. Interestingly, susceptibility to *Mycobacterium* infection is dependent on the polymorphism of the *SLC11A1* gene, encoding natural resistance-associated macrophage protein 1; this is also one of the genes associated with an increased risk of sarcoidosis [44]. *SLC11A1* gene polymorphisms have also been reported in many other diseases, including rheumatoid arthritis, irritable bowel syndrome, and multiple sclerosis. Also, the HLA alleles associated with sarcoidosis may increase the risk of tuberculosis, e.g., HLA-DRB1*04, or decrease it, e.g., HLA-DRB1*03 [44]. This association may be the reason for the co-occurrence of sarcoidosis with mycobacterial diseases, as well as the overlap of their clinical pictures.

#### 3.3.2. Microbiome of the Lower Respiratory Tract and Lung

It was long believed that the lungs and lower parts of the respiratory tract are free from microbial colonisation. This claim was strengthened by omission of those areas by the Human Microbiome Project and the tendency to regard the presence of microorganisms in samples of the upper parts of the respiratory tract as contamination [45,46]. Research into the microbiota of the lower respiratory tract was enabled by advancements in the collection of bronchoalveolar lavage fluid (BALF), e.g., by bronchoscopy, and the introduction of methods for culturing non-pathogenic genera; previous methods were focused on the identification of pathogens. Furthermore, advanced genetic methods, such as metagenomics, next-generation sequencing, and metabolomics enabled the detection of unculturable microorganisms, confirmed the existence of a rich and diversified microbiome throughout the whole respiratory system [45,46].

The composition of the microbiota of the lower respiratory tract is similar to those of its counterparts in the upper respiratory and digestive tracts. Their content is defined by a dynamic equilibrium between the influx of new microorganisms with inhaled air and their dispersion across the mucus, as well as their clearance by mucocilia, cough reflex, and immune cells [45,47]. The lungs are colonised mostly by the bacteria of four phyla, *viz. Bacteroidetes, Firmicutes, Proteobacteria*, and *Actinobacteria*, with the common genera being the bacteria *Pseudomonas*, *Streptococcus*, *Prevotella*, *Fusobacterium*, *Neisseria*, *Veillonella*, and *Haemophilus*, as well as the fungus *Aspergillus* [47,48,49]. A well-balanced lung microbiota can serve immunomodulatory functions, preventing allergic reactions and asthma as well as an excessive immune response against acute infections.

The composition and level of activity of the microbiome have been associated with multiple diseases, i.e., chronic obstructive pulmonary disease (COPD), asthma, and cystic fibrosis (CF). Lung dysbiosis can affect the activity of the immune system, directly or indirectly leading to the development of disease; however, some authors suggest that dysbiosis may occur as a result of a disease or its treatment [50]. Microbiome research is not only valuable for assessing the role of microorganisms in disease pathogenesis, but can also be used for earlier and more effective diagnosis of respiratory diseases and their prognosis [46,51].

Studies on the diversity of the respiratory tract microbiome in patients with sarcoidosis have not revealed any pulmonary dysbiosis, nor any significant difference in their microbial biodiversity compared to patients with interstitial lung disease [8]. Similar conclusions were reached by D’Argenio and colleagues [52], who report Bacteroidetes to be the most abundant group of microorganisms in the respiratory tract of patients with sarcoidosis and interstitial lung disease. However, both studies compared patients with respiratory diseases and neither included a group of healthy controls. In contrast, Gupta et al. [53] compared sarcoidosis with interstitial lung disease and COPD in a stable form and in exacerbations. Although these isolated microbiomes shared 11 taxa, as many as 11 amplicon sequence variants were unique to patients with sarcoidosis. The microbiome of the patients with sarcoidosis also demonstrated significantly lower α-diversity (understood as diversity within the analysed sample, through the assessment of richness and evenness) compared to those with stable COPD. Additionally, a greater abundance of *Actinobacteria*, *Proteobacteria*, *Corynebacterium*, and *Neisseria* was observed in patients with sarcoidosis than in patients with interstitial lung disease or COPD [53,54]. However, the β-diversity (which can be defined as dissimilarity between groups at a qualitative and quantitative level) analysis conducted by Gupta et al. [53] revealed an overlap of microbial taxa in respiratory tract ontocenosis communities between individual diseases [53,54].

Knudsen et al. [55] compared the lower airway microbiomes of patients with sarcoidosis with those of a healthy control group. The microbiome of the sarcoidosis patients was found to have significantly lower bacterial α-diversity than that of the healthy volunteers, with no differences in fungal α-diversity. Significant differences were also found at the β-diversity level [55]. An analysis of OTUs found *Atopobium* and *Fusobacterium* genera to be more frequently present in BAL samples from sarcoidosis patients compared to healthy controls, with an increased abundance in *Veillonella* and *Streptococcus* present in the latter [56]. Accordingly, Zimmermann et al. [56] suggested that *Atopobium* spp. and *Fusobacterium* spp. may influence the development of sarcoidosis; a deeper analysis showed that *Atopobium* spp. reached its greatest abundance in patients with stage I and II sarcoidosis, and *Fusobacterium* spp. in stage II and III; this indicates that disturbances in the species composition and abundance of the microbiome are associated with sarcoidosis development. Conversely, some evidence suggests that disturbances in the respiratory microbiome may also occur in response to long-term treatment with inhaled corticosteroids [56]. *Fusobacterium* colonisation has also been associated with many other diseases, including endometriosis and cancer [57,58]. This may indicate that these bacteria play a significant role in the development of health disorders, or that their opportunistic nature exploits pathological changes occurring in the body to increase their numbers.

#### 3.3.3. Gut–Lung Axis

**Figure 2:**
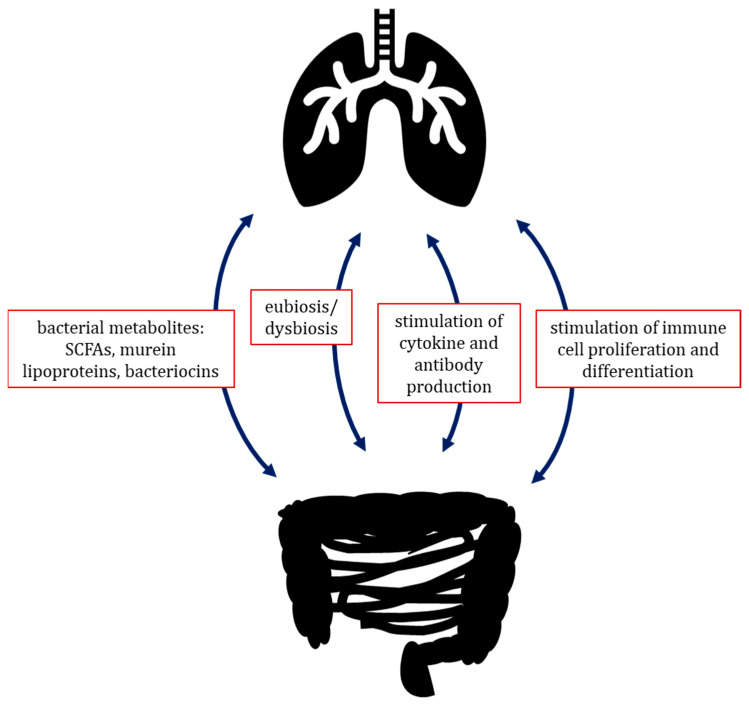
Lung–gut axis—hypothetical bidirectional signalling pathway between lung and gut microbiota. The proposed mechanisms of action of the lung–gut axis are based on imbalances in the intestinal and/or respiratory microbiota (eubiosis/dysbiosis), production of bioactive metabolites by microorganisms, stimulation of immune cell proliferation by microorganisms, and production of cytokines and antibodies by leukocytes as a result of stimulation by microbiota components.

Aside from their localised influence on surrounding tissues, the microorganisms inhabiting a certain compartment of the body are also able to influence remote areas, such as with the extensively studied gut–brain axis. The microbiota–gut–brain axis is defined as a bidirectional communication between the gut ontocenosis and the human nervous system. The communication system consists of several pathways, including the autonomic nervous system (ANS), the enteric nervous system (ENS), and the hypothalamic–pituitary–adrenal (HPA) axis, which transmit information from gut microbes to the brain and vice versa [59]. A recently discovered and promising analogue is the gut–lung axis, which is driven by interactions between gut microbiota and the host immune system, which are mediated by the circulatory system [60]. Some microbial metabolites, such as SCFAs and murein lipoproteins, stimulate the production and release of cytokines and antibodies by cells of the host, and influence the proliferation and differentiation of immune cells, as well as their activity. In addition, gut microbiome–host interactions are also able to influence the pulmonary immune system by immune cells migrating between individual lymphoid organs and tissues, such as Mucosa-Associated Lymphatic Tissue (MALT); some bacterial metabolic products can transfer to other organs, including the lungs, via the bloodstream, directly impacting the immune cells that reside there. Some research suggests that the gut–lung axis is bilateral: gut dysbiosis may lead to pulmonary infection and chronic diseases, and lung dysbiosis also has an effect on gut microbiota (Figure 2) [51,61,62].

Gastrointestinal dysbiosis may also lead to the development of sarcoidosis; Farahat et al. [63] attributed this to disturbances in the immune system, which could promote granuloma formation via the activation and secretion of various pro-inflammatory cytokines [63]. A recent study found significant differences in the gut microbiome between healthy people and sarcoidosis patients, suggesting that sarcoidosis may be linked to the presence of *Cereibacter sphaeroides* [64]. A study of the gut microbiota of sarcoidosis patients by Lee et al. [64] identified increased levels of three clusters related to heme biosynthesis, which translated into general immune activation, monocyte activity, and heme-related inflammation. It also found a positive correlation between these clusters and *C. sphaeroides*, capable of heme synthesis. The authors suggest that heme-related inflammation may be responsible for granuloma formation in sarcoidosis [64]. However, this is still an open issue that requires further detailed analysis.

#### 3.3.4. Blood Microbiome Dysbiosis

In recent years, greater scientific attention has been paid to the blood microbiome, i.e., the presence of living microorganisms in the blood. While it typically consists of small numbers of transient cells translocated from other ontocenoses in healthy people, dysbiotic blood microbiomes are characterised by elevated numbers of persistent microorganisms. Such dysbioses have been observed in, i.e., cardiometabolic diseases, cancers, and liver diseases to respiratory diseases, kidney dysfunction, immune and inflammatory disorders, and pregnancy complications, all of which are characterised by complicated aetiology [9,65].

Alterations in the diversity, richness, and composition of the blood microbiome have also been observed in the case of sarcoidosis. Dysbiosis of the blood microbiome may influence the progression of sarcoidosis by disrupting the immune system or metabolism, or by increasing intestinal permeability. One of the few studies examining the blood microbiome found greater α-diversity in sarcoidosis patients compared to healthy individuals, as well as differences at the β-diversity level, especially for the genera *Veillonella*, *Prevotella*, *Cutibacterium*, *Corynebacterium*, and *Streptococcus* [10]. However, the issue has been rarely studied, mainly due to the significant difficulty associated with blood testing, which is particularly subject to contamination.

On the other hand, it is very difficult to determine the composition of a *natural* or *common* blood microbiome. Based on the taxonomic analysis of the blood microbiota of 9770 healthy subjects, it was found that only 5% of participants shared the same species, and that the blood ontocenosis did not demonstrate any taxonomic similarity with other ontocenoses [66]. The term *ontocenosis* corresponds to the biocenosis at the level of microorganism habitats located in individual sites within the macroorganism [67]. However, research indicates that no common core blood microbiome is shared by the entire human population. This may suggest that microorganisms in the bloodstream are a transitional biota resulting from translocation from other ontocenoses [66].

#### 3.3.5. The Mycobiome in Sarcoidosis

While most research on the human microbiota has focussed on bacteria, some authors nevertheless emphasise the significance of the fungal mycobiota as a part of the microbiota. Research indicates the existence of a diversified mycobiome composed mainly of *Cladosporium*, *Eurotium*, *Penicillium*, and *Aspergillus* genera in the lung [68]. Indeed, greater species abundance and diversity has been noted in patients with certain pulmonary diseases, such as asthma, COPD, pneumonia, and CF, with the most common finding being an increase in the presence of *Aspergillus* spp. [49,69].

Few studies have been performed on the role of fungi in the etiopathogenesis of sarcoidosis and even fewer have been carried out at the mycobiome level. Despite this, higher numbers of *Aspergillus* and *Cladosporium* spp. have been detected in lymph node biopsies, BALF, Kveim reagent, and fresh granulomatous spleen samples from sarcoidosis patients; however, the findings were inconsistent between different sets of samples [70]. Also, significant differences in both the bacterial and fungal microbiome have been reported between stable sarcoidosis patients and healthy controls, with a high abundance of *Aspergillus* spp. in the lungs of sarcoidosis patients, and *Candida* spp. being more abundant in healthy people [55].

Greaves et al. [71] indicated that *Aspergillus nidulans* may have a role in the activation of CD4+ T lymphocytes. *A. nidulans* is widespread in the natural environment, and its spores are present the air, from where they are easily inhaled into the respiratory tract. Patients with sarcoidosis, presenting as Löfgren’s syndrome (LS), have demonstrated stimulation of CD4+ T cells by the *A. nidulans* antigen, albeit dependent on the HLA-DRB1*03 allele. The lymphocyte response may be influenced by NAD-dependent protein deacetylase, an intranuclear histone-interacting protein involved in transcriptional silencing [71]. In addition, it cannot be ruled out that epitopes from other fungal species will trigger similar activation of the T cell response. At present, very little is known about the connection between the influence of the fungal microbiota and the development of sarcoidosis, but this is a promising direction of research.

## 4. Summary

Due to its complex nature, the etiological and pathogenic mechanisms underlying sarcoidosis remain poorly understood. Furthermore, simply identifying these factors is not enough to create a complete picture of the disease; a greater amount of diversified data is needed to fully understand its etiopathogenetic mechanisms, and thus, determine the risk of developing sarcoidosis. Identification of factors triggering sarcoidosis and understanding their mechanisms of action would also improve the diagnostic process, replacing the current diagnosis of exclusion, as well as causal treatment. Furthermore, the epidemiological diversity observed between geographic regions and ethnic groups highlights the need for studies on different populations. Therefore, despite the accumulation of evidence highlighting the role of the human microbiota in health and in immune-mediated diseases (IMDs), further research is needed to elucidate the mechanisms by which microbial dysbiosis contributes to the pathogenesis of sarcoidosis.
